# Clinical Implications of Uric Acid in Heart Failure: A Comprehensive Review

**DOI:** 10.3390/life11010053

**Published:** 2021-01-14

**Authors:** Marko Kumrić, Josip A Borovac, Tina Tičinović Kurir, Joško Božić

**Affiliations:** 1 Department of Pathophysiology, University of Split School of Medicine, 21000 Split, Croatia; kumricjudo@gmail.com (M.K.); jborovac@mefst.hr (J.A.B.); tticinov@mefst.hr (T.T.K.); 2 Institute of Emergency Medicine of Split-Dalmatia County (ZHM SDZ), 21000 Split, Croatia; 3 Department of Endocrinology, University Hospital of Split, Spinčićeva 1, 21000 Split, Croatia

**Keywords:** uric acid, heart failure, biomarkers, OXIDATIVE stress

## Abstract

Affecting more than 26 million people worldwide and with rising prevalence, heart failure (HF) represents a major global health problem. Hence, further research is needed in order to abate poor HF outcomes and mitigate significant expenses that burden health care systems. Based on available data, experts agree that there is an urgent need for a cost-effective prognostic biomarker in HF. Although a significant number of biomarkers have already been investigated in this setting, the clinical utility of adding biomarker evaluation to routine HF care still remains ambiguous. Specifically, in this review we focused on uric acid (UA), a purine metabolism detriment whose role as cardiovascular risk factor has been exhaustingly debated for decades. Multiple large population studies indicate that UA is an independent predictor of mortality in acute and chronic HF, making it a significant prognostic factor in both settings. High serum levels have been also associated with an increased incidence of HF, thus expanding the clinical utility of UA. Importantly, emerging data suggests that UA is also implicated in the pathogenesis of HF, which sheds light on UA as a feasible therapeutic target. Although to date clinical studies have not been able to prove the benefits of xanthine oxidase in HF patients, we discuss the putative role of UA and xanthine oxidase in the pathophysiology of HF as a therapeutic target.

## 1. Introduction

With a worse prognosis than breast cancer in women and bladder cancer in men, heart failure (HF) represents a major global health problem [1,2]. This complex clinical entity, commonly defined as the inability of a heart to fulfil required metabolic demands and the perfusion of organs and tissues due to structural or functional cardiac abnormalities, is actually the most common cause of hospitalization after normal delivery, affecting more than 26 million people worldwide [3,4]. Major population studies reported stable incidence but an increase in HF prevalence and only a slight decrease in HF-related mortality in various populations, with a rather intriguing twist: a trend of a slight rise of HF-related mortality recently [5,6,7,8]. The observed increase in prevalence with stable incidence could be explained by the ageing population and improvements in HF treatment [2]. However, this rise will inevitably cause further increases in hospitalization rates and consequently, health care expenditures. Based on available data, experts agree that there is an urgent need for a cost-effective prognostic biomarker in HF. A significant number of biomarkers have already been investigated in the setting of HF [9]. So far, natriuretic peptides, cardiac troponin, and recently, soluble suppression of tumorigenicity-2 (sST2) have emerged as useful biomarkers indicated in HF diagnosis and risk stratification/prognosis [10]. In light of existent evidence, the Heart Failure Association of the European Society of Cardiology (ESC) consensus statement currently suggests a multi-marker approach that includes the above-noted biomarkers [9].

Owing to limited clinical applicability and low precise risk stratification of HF prognostic biomarkers, further research is needed in order to abate poor HF outcomes and mitigate significant expenses that burden health care systems. Specifically, in this review we focused on uric acid (UA), a purine metabolism detriment whose role as a cardiovascular risk factor has been exhaustingly debated for decades [11]. Apart from the prognostic role of UA, incorporated into a prognostic model recognized by the latest ESC guidelines, relevant heart failure societies at this moment do not formally address the issue of hyperuricemia in the context of heart failure diagnosis, prognosis, and treatment [3,12,13]. Of important note, emerging data from clinical studies suggest that aside from the well-established prognostic role in HF, UA is also implicated in the pathogenesis of HF, which sheds light on UA as a feasible therapeutic target [14]. Nevertheless, a larger body of evidence is needed to support these findings.

## 2. Underlying Molecular Mechanisms of HF Development

Virtually any disease or defect that impairs heart structure or function can subsequently lead to HF development. Although most commonly caused by coronary artery disease (CAD), unregulated diabetes, and hypertension, a palette of other etiologic factors, both intra- and extracardiac, can induce HF development [15]. 

In multiple pathophysiological pathways that are operative in HF, such as myocardial necrosis, upregulation of the renin-angiotensin-aldosterone system (RAAS), overt activation of the sympathetic nervous system, and endothelial dysfunction, a recently recognized pathologic process of endothelial-to-mesenchymal transition (EndoMT) emerged as a potent pathobiological driver of pro-fibrotic signaling pathways in HF, thus leading to myocardial fibrosis and adverse ventricular remodeling [16,17,18,19,20,21,22,23]. EndoMT is a dynamic shift in endothelial cell phenotype toward mesenchymal cells such as myofibroblasts, smooth muscle cells, and osteoblasts [24]. EndoMT-mediated fibrosis seems to be driven mainly by TGF-β via SMAD-2/3/4 and the Slug signaling pathway [25,26]. Hence, it has been hypothesized that EndoMT represents an integrative pathophysiological crosstalk between inflammation and fibrosis, making it a potential therapeutic target in HF [27]. Apart from being implicated in cardiac fibrosis, recent evidence indicates that EndoMT plays a role in several cardiac pathologies, including pulmonary artery hypertension, atherosclerosis, endocardial fibroelastosis, and valvular heart disease [28,29,30,31,32].

In 2013, Paulus and Tschöpe elaborated a pathophysiological model of HF with preserved ejection fraction (HFpEF), which proposes that highly prevalent co-morbidities of HF such as ageing, diabetes mellitus, metabolic syndrome, salt-sensitive hypertension, atrial fibrillation (AF), anemia, chronic obstructive pulmonary disease, and especially obesity exert their detrimental effects on the heart via endothelium of coronary microcirculation, which, as hypothesized, acts as a sort of central processing unit and transfers damage to the heart [33]. According to this model, the mentioned comorbidities induce a systemic pro-inflammatory state and thereby stimulate endothelial cells on reactive oxygen species (ROS) production [34]. Consequently, ROS trigger cardiomyocyte autophagy, apoptosis, or necrosis and reduce nitric oxide (NO) bioavailability, leading to endothelial dysfunction [33]. Importantly, ROS-mediated impaired nitric oxide-cyclic guanosine monophosphate-protein kinase G (NO-cGMP-PKG) signaling also leads to a rise in the resting tension of cardiomyocytes (i.e., myocardial stiffness) via hypophosphorylation of titin [35,36,37,38]. 

## 3. Molecular Mechanisms by Which UA Is Implicated in Pathophysiology of HF

UA is the end product of both dietary and endogenous purine metabolism in humans [39]. Due to the loss of uricase, an enzyme responsible for UA conversion into allantoin, humans are exposed to >50 times greater serum uric acid (SUA) concentrations than other mammals, making them susceptible to hyperuricemic repercussions [40]. Although from an evolutionary point of view the loss of uricase may have provided a survival advantage by amplifying the effects of fructose to enhance fat stores and by increasing blood pressure in response to salt, the absence of uricase gene expression, often referred to as “thrifty,” may have exhibited a range of detrimental effects on modern humans owing to the change in diet [41]. As a matter of fact, Neel et al. hypothesized that the loss of uricase could at least in part explain the current epidemic of obesity and diabetes [42]. Another evolutionary advantage of UA was proposed by Ames et al., who demonstrated that UA is a powerful scavenger of free radicals [43]. Figures suggest that UA contributes as much as 60% of free radical scavenging in human serum [44]. Moreover, systemic administration of UA increases plasma antioxidant capacity both at rest and after exercise in healthy volunteers [45,46].

Nevertheless, the biological effects of UA regarding oxidative stress are rather confounding. Unlike the antioxidant effects that UA exerts in extracellular, hydrophilic milieu, intracellularly it imposes detrimental effects, acting as a pro-oxidant [47]. Multiple experimental studies demonstrated that UA stimulated ROS creation in various cells, including endothelial cells, vascular smooth muscle cells (VSMCs), hepatocytes, and renal tubular cells, each with a set of repercussions. In endothelial cells it results in decreased NO bioavailability and inhibited cell migration and proliferation, whereas in hepatocytes it results in intracellular fat accumulation [48,49]. Furthermore, UA activates pro-inflammatory pathways and stimulates cell proliferation in VSMCs, stimulates EndoMT in renal tubular cells, and supports insulin resistance by generating oxidative stress in adipocytes [50,51,52]. Findings that implicate the pro-oxidative activity of UA are further substantiated by the protective effects of probenecid, an inhibitor of the organic anion transporter, which blocks the entry of UA into the cells and ameliorates oxidative stress [51]. Kang et al. tried to elucidate this peculiar dual role of UA in oxidative stress by the presence of an unrecognized molecular switch that controls the role of UA acting as a pro-oxidant or as an anti-oxidant [47]. Regarding the direct effects of UA on cardiomyocytes, multiple studies demonstrated that hyperuricemia inhibits myocardial cell activity by activating the extracellular signal-regulated kinase (ERK)/P38 signaling pathway through oxidative stress in vitro and induces cardiomyocyte apoptosis through the activation of calpain-1 and endoplasmic reticulum stress in rats [53,54,55]. Conversely, a study in healthy men showed that acute exposure to high levels of UA had no effect on hemodynamic variables, basal forearm blood flow, or nitric oxide-dependent endothelial function, implying that UA does not impair cardiovascular function [40]. Taken together, the direct effects of UA on the heart still remain quite ambiguous.

The last two steps of purine metabolism are catalyzed with xanthine oxidase (XO) [56]. The mentioned organic chemical reactions catalyzed by XO also generate free radicals as a byproduct [57]. Interestingly, XO was actually the first identified biological system to produce ROS and is in fact one of the strongest known sources of ROS production in human physiology [58]. In physiological milieu, XO-derived reactive oxygen species may have favorable effects, such as modulation of systemic redox balance and a line of defense against bacterial infections [59]. Conversely, overexpression of XO could have ROS-mediated detrimental effects such as endothelial function, inflammatory activation, mitochondrial damage, or impaired cardiac contractility, all of which are commonly seen in HF. Since the involvement of ROS in the development of HF has been well documented, multiple authors investigated the role of XO in HF pathophysiology in both animal and human studies [60]. XO upregulation in HF could be explained by commonly observed events in HF, such as hypoxia, increased catabolism, cell death, and insulin resistance, which lead to purine degradation and a subsequent increase in substrate supply [61,62]. In line with this, as demonstrated by multiple authors, a direct assessment of enzyme activity showed that XO activity was extremely upregulated (up to tenfold) in HF [63,64,65]. Studies suggest that XO is involved in HF development via endothelial dysfunction, myocyte apoptosis, and cardiac mechano-energetic coupling. Endothelial dysfunction is a direct result of an increase in the production of ROS as a consequence of XO upregulation in HF, as we noted in the previous section [66]. Additional evidence to support this notion is that the administration of allopurinol, a well-known XO inhibitor, improves endothelial dysfunction while reducing markers of oxidative stress among patients with HF [67]. In addition, Leyver et al. reported an inverse relationship between SUA and VO_2_ max and a positive correlation between UA levels and minute ventilation/carbon dioxide production (VE/VCO_2_), both of which suggest that increased SUA concentrations may reflect an impairment of the oxidative metabolism with consequent exercise intolerance in HF [68]. Apart from the abovementioned deleterious effects, XO upregulation is associated with increased filling pressures in systolic HF, diastolic dysfunction, and cachexia [69,70,71]. XO also emerged as a critical factor in upregulating myocardial apoptosis, a central feature in the progression of HF [72]. Finally, studies suggest that XO impedes the mechano-energetic uncoupling of the heart via crosstalk with cardiac NO signaling pathways. Mechano-energetic coupling is a phenomenon in a failing heart that implies that despite significantly impaired left ventricular LV work, the oxygen consumed for myocardial contraction remains relatively unchanged, resulting in a decrease in the mechanical efficiency of contractions [73]. It has been demonstrated that the administration of allopurinol in dogs with HF decreases oxygen consumption and increases myocardial contractility at both rest and exercise, as well as in response to the stimulation of dobutamine, all of the effects being limited to a failing heart [74,75]. In concordance with animal studies, Cappola et al. demonstrated that allopurinol administration can improve myocardial efficiency by decreasing oxygen consumption without simultaneous impairment in cardiac function [76]. Interestingly, these effects were abrogated by being blocked by N(G)-monomethyl L-arginine (L-NMMA), an NO synthase inhibitor, implicating the importance of NO signaling pathways in this process [75]. 

**Figure 1:**
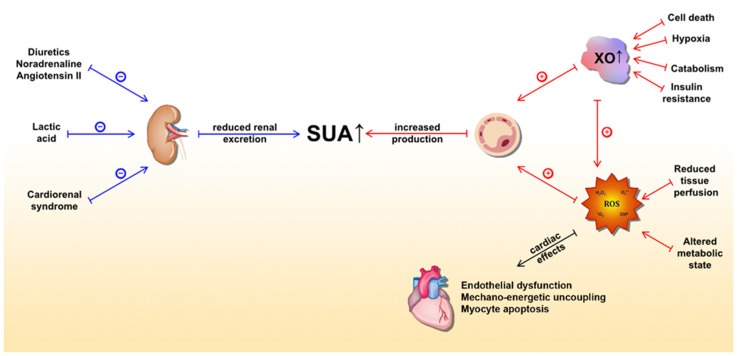
Underlying molecular mechanisms of serum uric acid (SUA) elevation in heart failure and detrimental effects of xanthine oxidase activity (mediated by ROS) on the heart. The blue lines represent mechanisms that lead to a reduction in UA excretion, whereas the red lines represent mechanisms leading to increased UA production. Abbreviations: SUA: serum uric acid; XO: xanthine oxidase; ROS: reactive oxygen species.

In the HF setting, elevated SUA levels are owed to at least two distinct mechanisms (Figure 1). The first is increased production and the latter is reduced excretion of UA. The former is caused by both a substantial increase in XO activity and increased oxidative stress, which arises from reduced tissue perfusion and altered metabolic state [56,72]. Conversely, multiple mechanisms lead to reduced kidney UA excretion. Functional renal impairment, as a part of cardiorenal syndrome, leads to decreased UA excretion in the kidney [77,78]. Furthermore, diuretics, which are widely prescribed to HF patients, lead to substantial loss of water and salt, thus stimulating proximal tubule reabsorption and a subsequent rise in SUA [79]. Other medications, such as noradrenaline and angiotensin II, can also promote hyperuricemia by stimulating UA tubular absorption [80]. In a state of impaired muscle perfusion and a consequent switch to an anaerobic metabolism such as HF, lactic acid plasma levels increase and lead to hyperuricemia by further mitigating UA renal excretion [81]. Ultimately, elevated UA itself can impair renal function, creating a positive feedback loop [82]. Of important note, patients with ischemic and non-ischemic HF show a similar distribution of SUA concentrations with respect to New York Heart Association (NYHA) classes [83]. This highlights the significant role of UA in HF independent of the presence of the metabolic syndrome, a common risk profile for ischemic heart disease, thus bringing further evidence that supports the notion that hyperuricemia is an intrinsic feature within the HF pathophysiology. Since SUA levels correlate with poor clinical outcomes of chronic HF (CHF) in a more evident manner among patients with impaired renal function, it seems that increased UA synthesis is a more significant contributor to the observed elevation of SUA in CHF than a reduction in UA excretion [84]. Conversely, Park et al. argued that cell death caused by ischemic insult or acute deterioration of renal function in acute heart failure (AHF) may be the dominant factor for hyperuricemia in that setting [85].

## 4. Clinical Implications of UA in HF

### 4.1. In the Acute Setting

To date, myriads of studies have been conducted regarding the clinical significance of UA in both AHF and CHF. A recent meta-analysis suggested that high SUA levels independently predicted all-cause mortality of patients with AHF, with hyperuricemia being associated with a 43% increase in all-cause mortality [86]. The same meta-analysis demonstrated that elevated SUA levels were associated with a 68% higher risk of a combined endpoint of death or readmission in AHF patients. Additionally, for each 1 mg/dL rise in SUA levels, the risk for all-cause mortality was increased by 11%, whereas pooled risk for a combined endpoint of death or readmission was increased by 12%. It is important to point out that none of the included studies showed either negative correlation or lack of correlation between SUA levels and AHF prognosis [85,86,87,88,89,90,91]. The main limitations of this meta-analysis were the low number of included studies (*n* = 10) and the fact that the use of diuretics, important regulators of UA excretion, was not clearly defined in the individual studies. The combination of UA and N-terminal pro-brain natriuretic peptide (NT-ProBNP) levels appears to be even more useful in AHF prognosis, as Park et al. demonstrated that a combination of the two is a better independent predictor for short-term outcomes in this setting than either of the markers alone [85]. In our single-center study that included 300 patients with AHF, we found that SUA levels were an independent predictor of all-cause mortality during the one-year follow-up and SUA levels >450 μmol/L conferred a 1.66 hazard ratio (95% CI 1.31–2.56, *P* < 0.001) for death [92]. Thus, it was included as a variable in the S2PLIT-UG risk score developed to estimate the one-year likelihood of mortality in AHF. By assessing in-hospital and long-term mortality in subjects with AHF from the Acute HEart FAilure Database (AHEAD) registry patients with AHF, Malek et al. demonstrated that elevated SUA levels and documented allopurinol therapy were associated with increased in-hospital and long-term mortality, with allopurinol not being a cause but rather the surrogate identifier of the subjects at risk for adverse outcomes [93]. Overall, it seems that SUA could be used as an adjunctive biomarker of poor prognosis in AHF, since its predictive role is independent of traditional prognostic determinants [94]. In line with this, a recent multicenter study included SUA levels greater than 7.2 mg/dL in the Preventing Re-hospitalization with TOLvaptan (Pretol) score, a novel scoring system that predicts the risk of rehospitalization for worsening heart failure [95].

### 4.2. In the Chronic Setting

About half of the patients suffering from HF with either preserved or reduced ejection fraction EF exhibit SUA concentrations above the upper reference limit [96,97]. The meta-analysis by Huang et al. and multiple studies that included patient follow-up demonstrated that elevated SUA levels are associated with increased incidence of CHF [98,99,100,101,102,103]. Huang et al. demonstrated that for each 1 mg/dL rise in UA, the odds of HF development increased by 19%, whereas results from the Framingham Offspring Cohort Study indicate that HF incidence rates were about sixfold higher among those at the highest quartile of SUA (>6.3 mg/dL) in comparison to those at the lowest quartile (<3.4 mg/dL) even after adjustment for confounding factors [100,101]. The correlation between gradual increase of SUA and HF incidence was additionally demonstrated by data extraction from the AMORIS study that included 417,734 men and women and from the Cardiovascular Health Study, where an increase of 1 mg/dL in SUA conferred a 12% increase in risk of new HF [99,104]. Among patients treated with antihypertensives, there are conflicting reports regarding the association between SUA and the risk of HF development [98,105]. In a recent study (British Regional Heart Study), Wannamethee et al. showed that male patients on antihypertensive treatment with SUA levels >6.9 mg/dL had a twofold higher risk of HF in comparison to those on treatment with levels <5.9 mg/dL but importantly, there was no difference among patients that were not treated with antihypertensives [106]. The latter raised doubts about the significance of UA in CHF pathophysiology.

The most widely discussed aspect of the role of UA in HF is in regard to the prognosis of patients with CHF. An important notion concerning prognostic studies is that older studies (before 2010) rarely adjusted variables relevant to the association of UA and adverse outcomes of HF patients [100]. The aforementioned meta-analysis by Huang et al. which included 28 studies reporting on HF outcomes, showed that UA is a predictor of all-cause mortality and CV mortality, whereas the combined incidence of death or cardiac events in HF patients did not reach statistical significance [100]. In fact, for every 1 mg/dL rise in SUA, all-cause mortality was greater by 4%. The authors also performed subgroup analysis on all-cause mortality by study design, sample size, adjustment or not, HF type, ethnicity, and duration of follow-up. All of the selected subgroups besides retrospective study design confirmed the correlation of UA serum concentrations and all-cause mortality among HF patients. These results are in accordance with a previous meta-analysis by Tamariz et al. that established a linear association between SUA and all-cause mortality above UA levels of 7 mg/dL and with several other large prospective studies with long follow-ups [94,107,108,109]. The same linear increase beyond SUA of ≥7 mg/dL, as well as poor long-term survival and increased risk of CV hospitalization in patients with CHF, was also demonstrated in a recent post hoc analysis of the Gruppo Italiano per lo Studio della Sopravvivenza nella Insufficienza Cardiaca-Heart Failure (GISSI-HF) trial [110]. Of note, Misra et al. found that CHF decompensation rates are positively associated with hyperuricemia (OR 1.67, 95% CI 1.21–2.32), whereas CHF recovery and diuretic discontinuation are associated with substantially lower odds of hyperuricemia (OR 0.21, 95% CI 0.08–0.55) [111]. Importantly, in a large pooled analysis of the Evidence for Cardiovascular Prevention from Observational Cohorts in Japan (EPOCH-JAPAN) study that included over 50,000 participants, Zhang et al. suggested a J-shaped relationship between SUA levels and CV mortality, with the highest quintile being associated with the largest CV mortality in both men and women and the middle levels with the lowest [112]. These findings could further substantiate the theory that due to the dual role of UA in oxidative stress, neither extremely low nor extremely high UA levels exert favorable effects on humans [47]. Recently, a post hoc analysis of the Metabolic Exercise Cardiac Kidney Index (MECKI) score database was conducted by Piepoli et al. [113]. By re-analyzing the study database, comprised of a large optimally treated HF with a reduced ejection fraction HFrEF patient population, researchers reached the conclusion that even though UA was associated with both CV and total death, it did not add prognostic power to the MECKI score in either the general HF population or in subgroups of patients with different HF severity (i.e., NYHA class and peak VO2). Interestingly, UA has been shown to predict CV death and total mortality in patients with less severe heart failure (i.e., NYHA class I or II) but not in patients with NYHA class III or IV. On the contrary, the Derivation study showed that SUA was the strongest prognostic variable in patients with severe CHF (NYHA class III or IV) and that it improved the prognostic power of the Heart Failure Survival Score (HFSS) [114,115]. In line with this, in a multivariate regression analysis SUA concentrations emerged as a significant predictor of NYHA functional class, independent of diuretic dose, age, body mass index, serum creatinine, alcohol intake, plasma insulin levels, and insulin sensitivity index [68]. The largest study (almost 100,000 participants) that failed to show positive association between elevated SUA levels and increased HF mortality in men and women, respectively, was the MJ Health Screening Cohort conducted in Taiwan [109]. Nonetheless, the study showed a weak positive association for both sexes combined. Several studies, including the latter, implied that elevated SUA levels in women were associated with a higher CV hazard ratio than that in men [109,112,116,117,118,119,120]. This may be because of the cardioprotective role of estrogen in women, with hyperuricemia being a hallmark of escape from estrogen-mediated CV protection [111,114]. Based on these considerations, UA has been incorporated in several risk assessment models for HF outcome prediction: the study of the effects of nebivolol intervention on outcomes and rehospitalization in seniors with heart failure (SENIORS) mortality risk model; the Metabolic, Functional, and Hemodynamic (MFH) staging system; and the Seattle Heart Failure Model [114,121,122]. Interestingly, Kim et al. also reported that SUA concentrations were independently a better predictor of poor outcomes than NT pro-BNP in HF patients [85].

### 4.3. Therapeutic Implications of UA in HF

Based on the evident pathophysiologic role of XO and UA in HF development, multiple authors tested the therapeutic potential of XO inhibition/UA reduction [11]. A panel of HF features were improved as a consequence of XO inhibition: myocardial mechanical and energetic efficiency, LVEF, cardiac remodeling, endothelial dysfunction, peripheral tissue perfusion, coronary flow reserve, cachexia, and plasma brain natriuretic peptide (BNP) levels [73,76,123,124,125,126,127,128,129]. In concordance, epidemiologic data suggested an association between allopurinol use and improved outcomes in HF patients with gout [130]. The use of uricosurics was also tested in several studies, yet they failed to exert comparable beneficial effects seen with XO inhibition [131,132,133]. Owing to promising results of preliminary studies, Hare et al. conducted the Oxypurinol Therapy for Congestive Heart Failure (OPT-CHF) trial, a randomized controlled trial (RCT), to study the hypothesized benefit of XO inhibition (oxypurinol) on mortality in CHF [134]. The study was not able to show a reduction in HF morbidity and mortality nor an improvement in quality of life. However, the subgroup analysis of patients with elevated UA levels (≥9.5 mg/dL) showed the expected favorable effect of XO inhibition. Since the latter observation was insufficient to confirm the utility of XO inhibition in HF treatment, an additional RCT was conducted recently [135]. Unlike the OPT-CHF trial, in which patients were included in the trial regardless of their baseline UA level, the Xanthine Oxidase Inhibition for Hyperuricemic Heart Failure Patients (EXACT-HF) trial enrolled patients with symptomatic HF and reduced LVEF (≤40%), markedly elevated SUA levels (≥9.5 mg/dL), and relatively well-preserved renal function (eGFR ≥ 20 mL/min). Patients were treated with allopurinol (target dose, 600 mg daily) or a placebo alongside their HF therapy and clinical outcomes were assessed at 12 and 24 weeks. The trial results were rather disappointing, as XO inhibition with high-dose allopurinol failed to improve clinical status, exercise capacity, quality of life, or LVEF at 24 weeks. Another XO inhibitor, febuxostat, was expected to exert an even stronger effect on XO inhibition than allopurinol [136,137,138]. However, reports concerning its use in HF patients were rather contradictory. In the Cardiovascular Safety of Febuxostat or Allopurinol in Patients with Gout (CARES) trial, all-cause mortality and cardiovascular mortality were higher with febuxostat in comparison to allopurinol and there was no difference between the two with respect to rates of adverse cardiovascular events [139]. Conversely, Cicero et al. demonstrated that febuxostat favorably affects cardiovascular mortality in comparison with allopurinol in elderly patients with mild-to-moderate HF [140]. However, no advantage or disadvantage of febuxostat to other SUA-lowering treatments (dominantly allopurinol) were demonstrated with respect to the onset of cardiovascular disease, yet febuxostat was associated with an increased risk of cardiovascular death in a meta-analysis by Cuenca et al. [141]. To sum up, although RCT results are discouraging given the conflicting data, future well-designed studies are required in order to rule out XO inhibitors as viable therapeutic agents in the treatment of HF.

## 5. Conclusions and Future Perspectives

Based on the available data it seems that UA has a wide set of feasible routine clinical applications in HF (Table 1). In AHF, particularly if combined with discharge NT-proBNP levels, high SUA concentrations could be used both as marker of poor prognosis with respect to mortality and as an indicator of increased risk for re-hospitalization in severe AHF. In patients with a high risk of developing CHF, SUA levels could be routinely measured, as this test is available in virtually any primary care laboratory (unlike NT-proBNP) and it contemporarily reflects an increase in risk of incident HF. Thus, in situations where secondary care is of limited availability, SUA levels could serve as an additional parameter for triage. On the other hand, due to a linear association between extremely high SUA levels and CV mortality, extremely high levels of UA in patients with diagnosed CHF should inform practice with respect to the increased mortality risk of these patients. Despite results of clinical trials that tested XO inhibitors in the treatment of HF being neutral and missing primary endpoints, we believe that further research is still warranted, as studies that showed no benefits enrolled only patients with HFrEF, whereas most of the studies that exerted positive results did not make a distinction between the HF phenotypes. Furthermore, it might be of greater importance to pharmacologically target other limbs of purine metabolism beyond sole XO inhibition in order to mitigate overt ROS production and dysfunctional NO-generating pathways.

## Figures and Tables

**Table 1:** Clinical applications of uric acid in heart failure.

Clinical Parameter	Clinical Significance	Clinical Setting	Supporting Evidence
CHF incidence	For each 1 mg/dL rise in UA there is a 12–19% increase in risk of new HF	General population [78]; general population >65 y [104]; heterogeneous population * [100]	AMORIS [99]; Cardiovascular Health Study [104]; Huang et al. [100]
	HF incidence rates are sixfold higher among those at the highest quartile of UA (>6.3 mg/dL) vs. the lowest quartile (<3.4 mg/dL)	General population [101]	Framingham Offspring Cohort Study [101]
CHF prognosis	For each 1 mg/dL rise in UA, all-cause mortality increases by 4%	Heterogeneous population * [100]	Huang et al. [100]
	Linear association between SUA and all-cause mortality above UA levels of 7 mg/dL	Patients with CHF [94,110]	Tamariz et al. [94]; GISSI-HF [110]
	J-shaped relationship between SUA levels and CV mortality	General population [112]	EPOCH-JAPAN [112]
	UA does not add prognostic power to the MECKI score	Patients with HFrEF [113]	Piepoli et al. [113]
	UA is the strongest prognostic variable in patients with severe CHF	Patients with CHF [114]	Derivation study [114]
	Elevated SUA levels in women are associated with a higher CV hazard ratio than that in men	General population [109,112,116,117]	EPOCH-JAPAN [112]; Chen et al. [109]; NHANES I [116,117]
	Part of prognostic risk models: SENIORS mortality risk model MFH staging system Seattle Heart Failure Model	Patients with CHF [114,119]; patients with HFrEF [120]	Derivation study [114]; Levy et al. [119]; Manzano et al. [120]
AHF prognosis	For each 1 mg/dL rise risk for all-cause mortality increases by 11% Pooled risk for combined endpoint of death or readmission increases by 12%	Patients with AHF [86]	Huang et al. [86]
	Positive correlation with increased in-hospital and long-term mortality	Patients with acute decompensated CHD or de novo HF [93]	Malek et al. [93]
	UA + NT-ProBNP combination is a better independent predictor for short-term outcomes in HF than either of the markers alone	Patients with AHF [85]	Park et al. [85]
	Predictive role of UA is independent of traditional prognostic determinants	Patients with AHF [94]	Tamariz et al. [94]
	SUA levels >450 μmol/L associated with 1.66-fold increase in risk of all-cause death in the AHF cohort of patients. Patients with mean SUA of 606 μmol/L or higher were at the highest risk of death. S_2_PLIT-UG score	Patients with AHF [92]	Borovac et al. [92]
	UA levels >7.2 mg/dL as a part of Pretol score, which predicts the risk of re-hospitalization for worsening HF	Patients with acute decompensated HF [95]	Takimura et al. [95]

* Meta-analysis included studies with AHF patients, CHF patients, and general population, respectively. Abbreviations: CHF: chronic heart failure; AHF: acute heart failure; UA: uric acid; AMORIS: Apolipoprotein MOrtality RISk study; MECKI: Metabolic Exercise Cardiac Kidney Index; MFH: metabolic, functional, and hemodynamic; GISSI-HF: Gruppo Italiano per lo Studio della Sopravvivenza nella Insufficienza Cardiaca-Heart Failure; EPOCH-JAPAN: Evidence for Cardiovascular Prevention from Observational Cohorts in Japan; NHANES I: National Health and Nutrition Examination Survey; HFrEF: Heart failure with reduced ejection fraction; CV: cardiovascular; NT-ProBNP: N-terminal pro-brain natriuretic peptide.

## Data Availability

The data presented in this study are available on request from the corresponding author. The data are not publicly available due to our further research in this field.
